# Inorganic nanoparticle‐integrated mesenchymal stem cells: A potential biological agent for multifaceted applications

**DOI:** 10.1002/mco2.313

**Published:** 2023-07-31

**Authors:** Juan‐Juan Zheng, Xin‐Chi Jiang, Yao‐Sheng Li, Jian‐Qing Gao

**Affiliations:** ^1^ Institute of Pharmaceutics College of Pharmaceutical Sciences Zhejiang University Hangzhou China; ^2^ Hangzhou Institute of Innovative Medicine College of Pharmaceutical Sciences Zhejiang University Hangzhou China; ^3^ Dr. Li Dak Sum & Yip Yio Chin Center for Stem Cell and Regenerative Medicine Zhejiang University Hangzhou China

**Keywords:** antitumor therapy, in vivo tracking, inorganic nanoparticles, mesenchymal stem cells, regenerative therapy

## Abstract

Mesenchymal stem cell (MSC)‐based therapies are flourishing. MSCs could be used as potential therapeutic agents for regenerative medicine due to their own repair function. Meanwhile, the natural predisposition toward inflammation or injury sites makes them promising carriers for targeted drug delivery. Inorganic nanoparticles (INPs) are greatly favored for their unique properties and potential applications in biomedical fields. Current research has integrated INPs with MSCs to enhance their regenerative or antitumor functions. This model also allows the in vivo fate tracking of MSCs in multiple imaging modalities, as many INPs are also excellent contrast agents. Thus, INP‐integrated MSCs would be a multifunctional biologic agent with great potential. In this review, the current roles performed by the integration of INPs with MSCs, including (i) enhancing their repair and regeneration capacity via the improvement of migration, survival, paracrine, or differentiation properties, (ii) empowering tumor‐killing ability through agent loaded or hyperthermia, and (iii) conferring traceability are summarized. An introduction of INP‐integrated MSCs for simultaneous treatment and tracking is also included. The promising applications of INP‐integrated MSCs in future treatments are emphasized and the challenges to their clinical translation are discussed.

## Graphical abstract

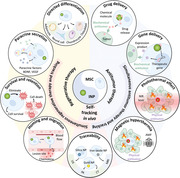

## 1 INTRODUCTION

Stem cell therapy is an innovative treatment strategy that restores damaged cells and tissues in the body by utilizing stem cells' unique capacities for self‐renewal and differentiation.^1^ Among them, mesenchymal stem cells (MSCs) are preferred due to their simplicity in separation and multiplication,^2^ multilineage differentiation potential,^3^ immunomodulation ability,^4^ tropism to inflammation site,^5^, ^6^, ^7^ and exemption from ethical issues.^8^, ^9^, ^10^ Since their first documented clinical application,^11^ MSC‐based therapies have received substantial research due to their therapeutic promise in many intractable diseases.^12^, ^13^, ^14^, ^15^, ^16^, ^17^, ^18^, ^19^, ^20^ Based on their applications, these MSC‐based therapies can be split into two main groups: those that directly exert therapeutic effects themselves or as vehicles for drug delivery. In the former category, MSCs are commonly applied as restorative biological agents in regenerative medicine.^21^, ^22^, ^23^, ^24^ In the case of the latter category, MSCs are primarily designed as lethal biological agents for antitumor therapeutics due to their inherent tumor‐tropic homing and migration capabilities.^25^, ^26^, ^27^, ^28^ However, a number of issues still limit the clinical use of MSC‐based therapies. For MSCs used in damage repair, low transplant and viability rates of implanted cells are major issues. The insufficient MSCs reserved in the lesion site would fail treatment.^29^, ^30^ As for MSCs applied in antitumor therapy, the low drug‐loading capability, especially for cytotoxicity drugs, limits their ability to kill tumors. And the drug loaded could adversely affect the function of cellular carriers, which further reduces the delivery efficiency. In addition, unclear in vivo fate is also a problem, making it difficult to resolve the treatment mechanism and ensure safety. Engineering of MSCs was often used to enhance the treatment effect and offer promising strategies to solve the above problems. For example, viral vectors, such as lentiviruses, can be used to genetically modify MSCs to overexpress B‐cell lymphoma‐2 and vascular endothelial growth factor (VEGF), aiming to enhance paracrine effects and improve the survival of MSCs.^31^ Yet viral vectors would increase concern about safety issues.^32^ And the viral modification mainly changes single protein expression,^33^ making it difficult to achieve therapeutic diversification.

Recently, the rapid development of nanotechnology opens a new door to address the primary difficulties with stem cell therapy.^34^ The distinctive characteristics of nanomaterials herald an almost limitless variety of applications. Different kinds of nanoparticles (NPs), such as lipid‐based NPs, polymeric NPs, and inorganic NPs, have been employed extensively in biomedical fields recently, including improved drug delivery, tissue engineering, disease detection, and medical imaging.^35^, ^36^, ^37^, ^38^, ^39^, ^40^ Each class of these NPs have distinct application areas based on their individual characteristics. For example, polymeric NPs allow for easy surface modification,^41^ while high biocompatibility is a prominent advantage of lipid‐based NPs. Although inorganic nanoparticles (INPs) have disadvantages in terms of biodegradability and payload flexibility compared with the other classes of NPs, they have higher variability and controllability in size, structure, and shape.^42^ Moreover, INPs have distinct qualities of physics, electricity, magnetism, and optics that depend on the base material.^43^, ^44^, ^45^ These qualities allow INPs to perform additional functions to satisfy various applications. First, INPs offer fascinating opportunities for simultaneous imaging and medication administration, as many of them, such as iron oxide NPs, silica NPs, and gold NPs, are also excellent contrast agents in addition to delivering drugs.^46^, ^47^, ^48^ Second, certain INPs, like gold NPs^25^ and iron oxide NPs, can generate hyperthermia to aid in treatment.^49^ Third, INPs allow for on‐demand medication release or magnetically tailored administration, due to their responsiveness to exterior stimulation such as ultrasound (US) for silica NPs, near‐infrared (NIR) light for gold NPs, or magnetic fields (MF) for iron oxide NPs.^50^, ^51^, ^52^

The integration of these INPs into MSCs holds the potential for achieving therapeutic diversification in stem cell therapy. Two methods for integrating INPs with MSCs are available: internalization into the cell or anchoring to the cell surface.^53^ INPs can be internalized by MSCs through nonspecific endocytosis or receptor‐mediated specific endocytosis.^54^, ^55^, ^56^ The latter is relied on the surface functionalization of INPs.^57^ In addition, some INPs that have protein or antibody surface modifications can be anchored to the cell surface via ligand–receptor interactions.^58^, ^59^, ^60^

Once integrated, the INPs can endow new functions to MSCs and facilitate MSC‐based therapies. First, INP integration can improve the migration, survival, paracrine, and differentiation properties of MSCs through enhanced drug‐loading capability or self‐biological effects, thus optimizing their therapeutic efficacy as restorative biological agents. Second, INP integration can make MSCs powerful tumor‐killing agents through the improved drug‐loading capability or self‐physical high thermal properties. Third, incorporating INPs as contrast agents can confer traceability to MSCs, enabling the in vivo location and viability tracking of MSCs, thus guiding precise transplantation, elucidating therapeutic mechanisms, and ensuring safety. In summary, the hybrid systems offer the benefits of both INPs and MSCs and can be tailored for specific biomedical applications.

**FIGURE 1:**
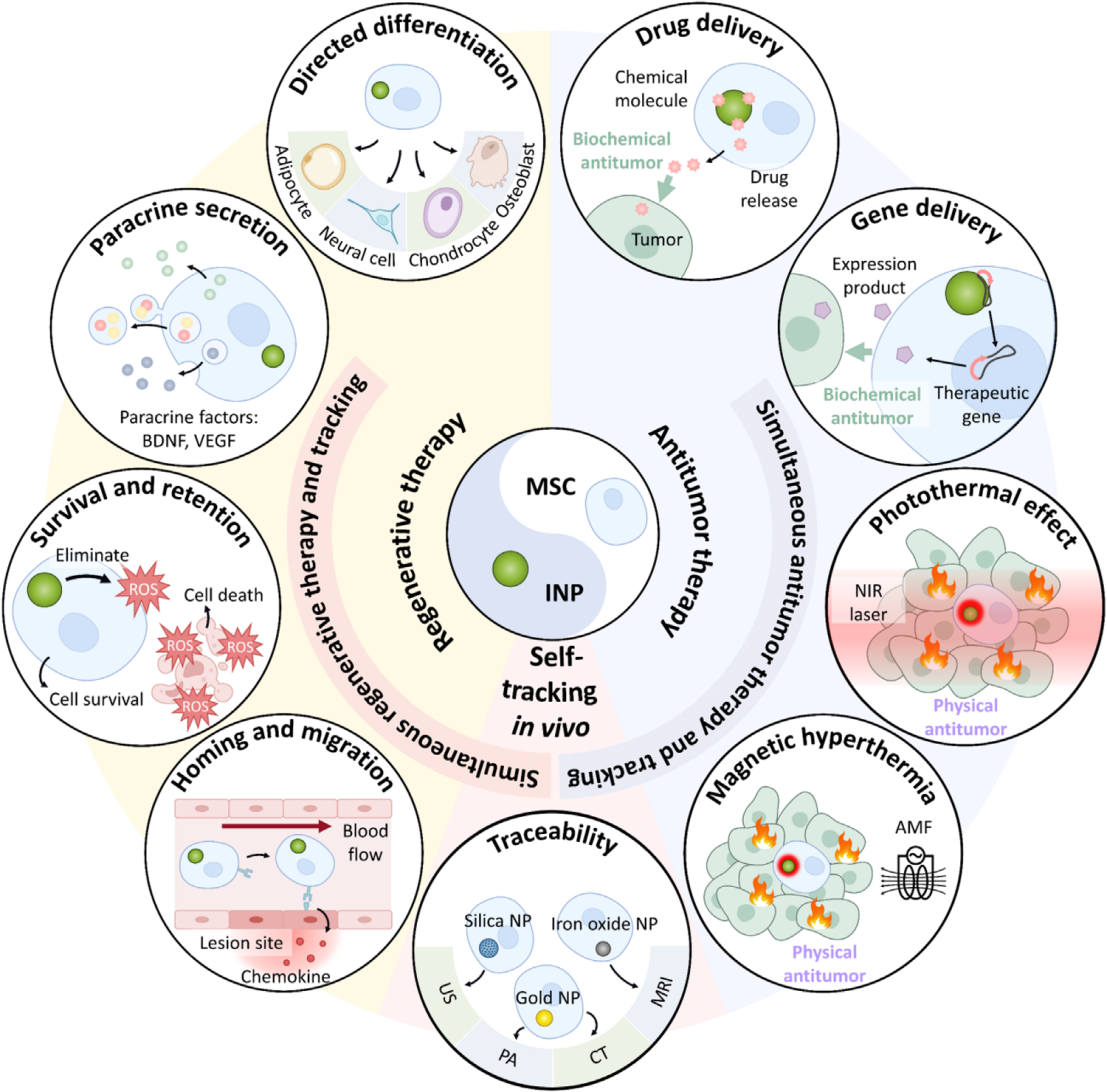
The scheme of potential applications and therapeutic mechanisms of INP‐integrated MSCs. INP‐integrated MSCs are a potential biological agent for multifaceted applications: regenerative therapy via the improvement of migration, survival, paracrine or differentiation properties, antitumor therapy through agent loaded or hyperthermia, self‐tracking in vivo through the traceability conferred by INP integration. Also, they can achieve the combination of multiple applications. *Abbreviations*: AMF, alternating magnetic field; BDNF, brain‐derived neurotrophic factor; CT, computed tomography; INP, inorganic nanoparticle; MRI, magnetic resonance imaging; MSC, mesenchymal stem cell; NIR, near‐infrared; NP, nanoparticle; PA, photoacoustics; US, ultrasound; VEGF, vascular endothelial growth factor.

Iron oxide NPs, silica NPs, and gold NPs cover a wide range of additional functions of INP systems, and numerous preclinical studies have demonstrated the beneficial effects of their integration for MSC‐based therapies.^61^, ^62^, ^63^ Other metals and metal oxides, including Ag, Pt, MnO_2_, CuO, and ZnO, also have broad applications in biomedical fields involving bioanalysis, inflammation mitigation, antibacterial, and cancer therapy.^64^, ^65^, ^66^, ^67^, ^68^, ^69^ However, fewer studies have integrated them with MSCs due to concerns about potential cytotoxicity and inadequate understanding of their interactions with cells.^70^, ^71^, ^72^ Considering the potential for future clinical translation, we discuss the most commonly utilized INPs‐integrated MSCs to serve as biological agents for multifaceted applications, specifically those composed of iron oxide, silica, and gold. This review first summarizes the current roles performed by the integration of INPs with MSCs (Figure 1), including (i) altering the properties of MSCs and improving their repair and regeneration capacity, (ii) empowering tumor‐killing ability through agent loaded or hyperthermia, and (iii) conferring traceability. An introduction of INP‐integrated MSCs for simultaneous treatment and tracking is also included. And we then emphasize the promising applications of INP‐integrated MSCs in future treatments and discuss the challenges to their clinical translation.

## 2 INP‐INTEGRATED MSCS FOR REGENERATIVE THERAPY

MSCs are hotspots in tissue healing and regenerative medicine. On the one hand, the multidirectional differentiation potential of transplanted MSCs can directly replenish damaged tissues. On the other hand, the paracrine effect of MSCs could help modulate the inflammation microenvironment and facilitate tissue repair.^73^ For optimal regenerative therapy, sufficient lesion site delivery and retention of MSCs as well as enough preservation of biological activity of MSCs for differentiation and paracrine secretion are needed. INP integration can improve the therapeutic ability of MSCs in several ways, including (i) increasing the homing and migration capacity of MSCs to the lesion site, (ii) improving the survival and retention of MSCs in the disease microenvironment, (iii) promoting beneficial paracrine secretion of MSCs, and (iv) enhancing the directed differentiation of MSCs, thus INPs are the ideal candidate to optimize MSC‐based regenerative therapy. Table 1 provides an overview of the pertinent contents.

**TABLE 1:** Summary of the current role performed by INPs in facilitating MSC‐based regenerative therapies.

Enhancement	Nanoparticle	Mechanism	Subsidiary	References
Homing and migration	PEGylated hollow gold NP	Upregulation of actin and microscopic proteins expression	/	^74^
	Endosome‐triggered iron‐ion‐releasing NP	Upregulation of CXCR4 expression	/	^75^
	Magnetic Fe_3_O_4_@PDA NP		/	^76^
	Ferrimagnetic iron oxide nanochain		/	^77^
	SPIO@PLL NP	Magnetic guidance	Magnet (0.39 T)	^78^
	SPIO@PEG NP		Magnet (0.3 T)	^79^
	SPIO@PLL NP		Magnetic system	^80^
	Fe_3_O_4_@PDA NP		Magnet	^81^
	Fe_3_O_4_@PDA NP		Magnet (1.2 T)	^82^
	Magnetic Fe_3_O_4_ NP		Magnet (1.2 T)	^83^
	SPION	Upregulation of CXCR4 expression; magnetic attraction	Magnet (0.32 T)	^84^
Survival and retention	TEMPO‐conjugated gold NP	ROS clearance via delivery of TEMPO	/	^85^
	CoPP‐loaded MSN	Upregulation of HO‐1 expression via delivery of CoPP	/	^86^
	Wnt3a‐loaded porous silica NP	Antioxidative stress via Wnt/β‐catenin signaling pathway	/	^87^
	Nanocomplex of Cu*_x_* O NPs and gold NPs encapsulated in oxidation‐sensitive dextran	ROS scavenging by responsive release of Cu*_x_* O NPs	/	^88^
Paracrine secretion	BDNF/mCherry fusion gene‐loaded gold NP	Enhancement of BDNF expression via gene delivery	/	^89^
	BDNF pDNA‐loaded ferrimagnetic iron oxide nanochain	Enhanced expression of VEGF via efficient gene delivery	/	^77^
	VEGF pDNA‐loaded cross‐linked iron oxide NP	Increase of VEGF production via sufficient gene transfection	MF (0.3 T, 15 min)	^90^
	Fe_3_O_4_@PDA NP	Regulation of essential proteins expression in the hippocampus	/	^91^
	Endosome‐triggered iron‐ion‐releasing NP	Upregulation of HIF‐1α expression via iron ion level	/	^75^
	Iron oxide NP	Enhancement of gap junctional crosstalk between MSCs and cardiomyoblasts via upregulation of CXCR4 expression	Coculture with cardiomyoblasts	^92^
Osteogenic differentiation	Gold NP	Activation of the P38 MAPK pathway	/	^93^
	Gold NP‐loaded hydroxyapatite composite	Activation of Wnt/β‐catenin signaling pathway	/	^94^
	Chitosan‐conjugated gold NP	Activation of Wnt/β‐catenin signaling pathway	/	^95^
	Iron oxide NP	Activation of canonical MAPK signaling pathway	/	^96^
	Silica NP	via the release of Si ions	/	^97^
	DMOG‐loaded mesoporous silica nanosphere	Activation of bone‐related gene and protein expression via the release of Si ions	Promotion of angiogenesis via delivery of DMOG	^98^
	Silver NP	Activation of autophagy	/	^99^
	Platinum NP	Possible impact on certain osteogenic signaling pathways	/	^100^
	BMP‐2‐derived peptide and Dex incorporated MSN	via the synergy of BMP‐2‐derived peptide and Dex	/	^101^
	Calcium phosphate‐coated and Sr‐incorporated MSN	via delivery of bioinorganic ions Sr or Zn	/	^102^
	BMP‐2 pDNA and Dex‐loaded functionalized MSN	Codelivery of osteogenic genes and drugs	/	^103^
	Peptide‐conjugated and miR‐26a‐loaded MSN	Promotion of osteogenesis‐related key growth factor expression	/	^104^
	MiR‐21‐conjugated silver NP and miR‐148b‐conjugated gold NP	Combinatorial delivery of miR‐21 and miR‐148b	/	^105^
Chondrogenic differentiation	Iron oxide‐based magnetic NP	Magneto‐mechanical stimulation	MF (6 mT) for 10 min every 2 h	^106^
Neural differentiation	PEG‐phospholipid encapsulated magnetite (Fe_3_O_4_) NP	Activation of CREB phosphorylation	ELF‐EMFs (1 mT, 50 Hz)	^107^
Adipogenic differentiation	PEI‐coated and C/EBP beta pDNA gene‐loaded gold NP	via delivery of genes encoding appropriate differentiation factors	/	^108^
Trilineage differentiation	HSA‐coated and FGF2‐conjugated iron oxide NP	via enhanced biological efficacy of FGF2	/	^109^

Abbreviations: BDNF, brain‐derived neurotrophic factor; BMP‐2, bone morphogenetic protein‐2; C/EBP, CCAAT/enhancer binding protein; CoPP, cobalt protoporphyrin; CREB, cAMP‐response element binding protein; Dex, dexmedetomidine; DMOG, dimethyloxallyl glycine; ELF‐EMFs, extremely low frequency electromagnetic fields; FGF2, fibroblast growth factor2; HAS, human serum albumin; HIF‐α, hypoxia inducible factor‐α; MAPK, mitogen‐activated protein kinase; MF, magnetic field; MSCs, mesenchymal stem cells; MSN, mesoporous silica nanoparticle; NP, nanoparticle; PDA, polydopamine; pDNA, plasmid DNA; PEG, polyethylene glycol; PEI, polyetherimide; PLL, poly‐l‐lysine; ROS, reactive oxygen species; SPIO, super paramagnetic iron oxide; TEMPO, 2,2,6,6‐tetramethylpiperidine‐N‐oxyl; VEGF, vascular endothelial growth factor.

### 2.1 INP integration increases homing and migration of MSCs

Sufficient lesion site delivery of stem cells is critical for efficacy. MSCs express specific C‐X‐C chemokine receptor type 4 (CXCR4) and other chemokine receptors (including CCR1, CCR2, CCR4, CCR7, etc.), that respond to growth factors, chemokines, and cytokines at the site of injury, which enable lesion site targeting of MSCs.^110^

**FIGURE 2:**
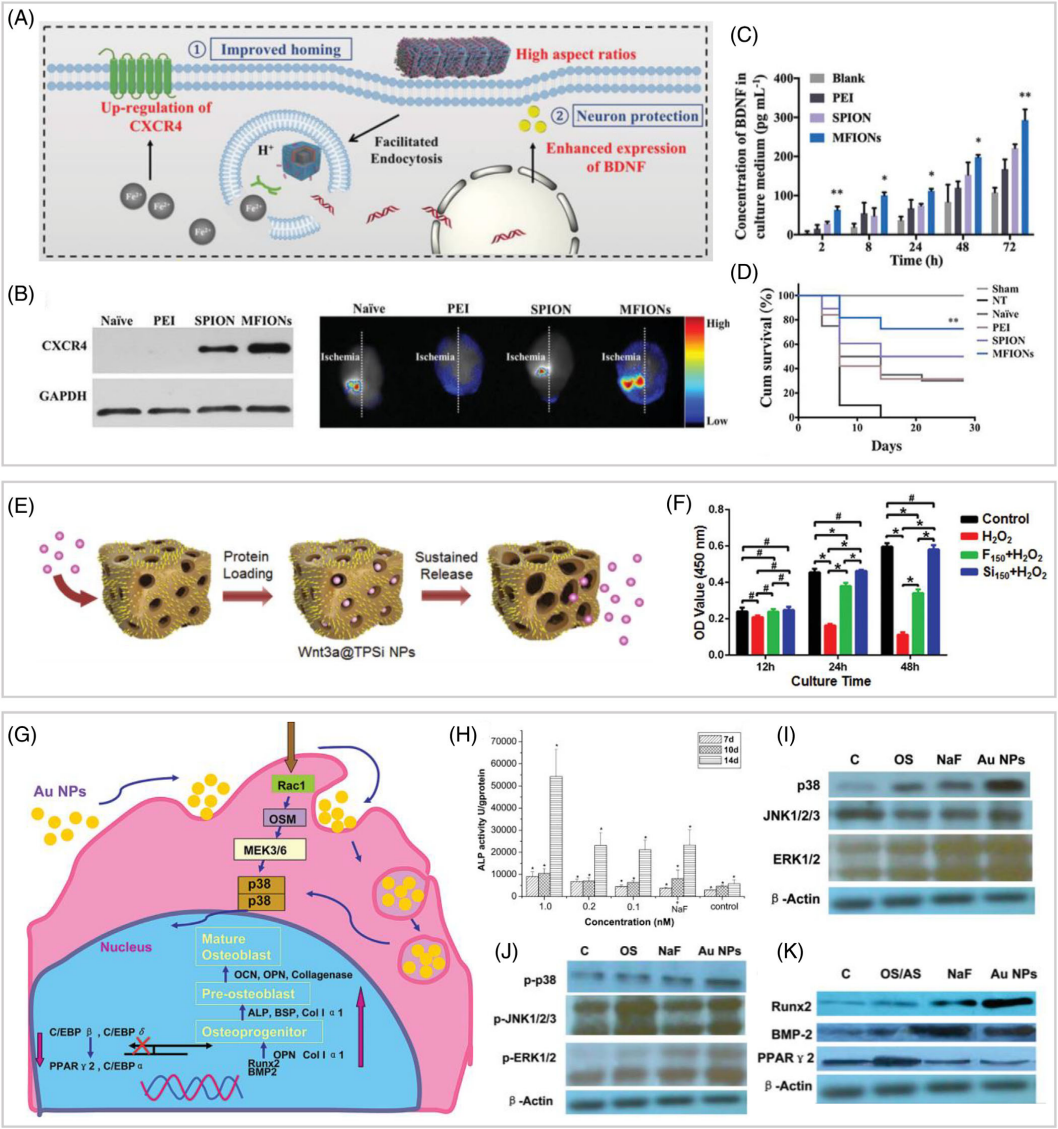
Representative examples of the beneficial role of INP integration in facilitating MSC‐based regenerative therapies. (A) Schematic illustration of the beneficial effects of magnetosome‐like ferrimagnetic iron oxide nanochain (MFION)‐integration on MSCs. (B) MFION‐integration significantly increased homing of MSCs to the ischemic hemisphere via the upregulation of CXCR4 expression. (C) MFION‐integration promoted the sustained secretion of BDNF from MSCs. (D) Treatment of MFION‐integrated MSCs significantly improved survival in mice with ischemic stroke injury. Reprinted with permission from Ref. 77, Copyright 2019 John Wiley & Sons. (E) Schematic illustration of the loading of Wnt3a and its achievable sustained release. (F) The integration of Wnt3a‐loaded porous silica NPs achieved more durable protection on labeled MSCs against H_2_O_2_. Reprinted with permission from Ref. 87, Copyright 2019 John Wiley & Sons. (G) Schematic illustration of the intracellular mechanism regulating osteogenic differentiation of MSCs by gold NP‐integration. (H) Gold NP‐integration significantly increased ALP activity in MSCs on a dose‐ and time‐dependent basis. (I) Gold NP‐integration induced activation of p38 MAPK pathway in MSCs. (J) Phosphorylation of p38 MAPK in MSCs by gold NP‐integration. (K) The upregulation of Runx2 and BMP2 protein expression in gold NP‐integrated MSCs. Reprinted with permission from Ref. 93, Copyright 2010 American Chemical Society.

INP integration can enhance the original migration capabilities of MSCs. It was found that the integration with gold NPs increased the content of actin and microscopic proteins in MSCs, and their migration ability was correspondingly enhanced.^74^ Targeting relevant signaling pathways is more advantageous in promoting the migration of MSCs than targeting the cytoskeleton. Some iron ion‐releasing NPs,^75^ including classical iron oxide NPs, were able to induce the upregulation of CXCR4 in MSCs. The enhanced CXCL12/CXCR4 axis then improved the enrichment of MSCs at the lesion site. in vitro migration assays showed that the migration capacity of MSCs internalized with iron oxide NPs increased by three times.^76^ Moreover, the integration of ferrimagnetic nanochains notably increased homing of MSCs to the ischemic hemisphere in the ischemic stroke mouse model (Figures 2A and B).^77^ The limitation of this strategy is that it requires the presence of the relevant chemokine at the lesion site.

Magnetic targeting technology is another strategy that could help to increase lesion site targeting of MSCs. The MSCs equipped with magnetic NPs, primarily based on magnetite, could respond to MF. By manipulating the external MF, they could be recruited to the target site. For instance, the application of MF notably improved the accumulation of magnetically labeled MSCs at the lesion site of spinal cord injury by a factor of three compared with the group without MF.^80^ The advantage of this strategy is that it is not dependent on molecules in the microenvironment of the lesion site. However, considering the convenience of external magnet arrangement, it is more suitable for lesion sites close to the superficial surface, such as the brain,^78^ heart,^79^ spinal cord,^80^, ^81^ pancreas,^82^ olfactory tissues,^84^ and burn sites in the skin.^83^

### 2.2 INP integration improves survival and retention of MSCs

Low survival and low retention are two key issues that affect the benefits of stem cell therapy. Stem cells targeted to the lesion site are easily damaged by toxic substances such as reactive oxygen species (ROS) in the inflammatory microenvironment and undergo apoptosis.^111^, ^112^ Therefore, they cannot exert a good therapeutic effect at the lesion site. NP‐modification can confer greater tolerance to stem cells.

INPs could serve as carriers for antioxidant drugs and be integrated into MSCs. This strategy has two main advantages. First, utilizing INPs could minimize the cyto‐concentration of free drugs while retaining their antioxidant capacity, as free antioxidant drugs would affect the viability of MSCs. For example, gold NPs could deliver ROS scavengers 2,2,6,6‐tetramethylpiperidine‐N‐oxyl to MSCs and reduce the level of H_2_O_2_‐induced overproduction of ROS in MSCs by 25%. Moreover, carrying ROS scavengers in INPs could reduce the inhibition on the osteogenic differentiation of MSCs.^85^

Second, the sustained drug‐release ability of NPs confers long‐term resistance to oxidative stress on integrated MSCs. in vitro experiments showed the cytoprotection of free Wnt3a only last for 12 h when MSCs subjected to H_2_O_2_ treatment. Among them, Wnt3a is a versatile protein that works through the Wnt/β‐catenin signaling pathway to defend cells against oxidative stress damage.^87^ Wnt3a‐loaded porous silica NPs achieved more durable protection, the viability of MSCs labeled with Wnt3a‐loaded porous silica NPs was less affected and is 70.3% higher compared with MSCs protected by free wnt3a when treated with H_2_O_2_ for 48 h (Figures 2E and F).

### 2.3 INP integration promotes paracrine secretion of MSCs

Paracrine secretion is an important approach for stem cells to exert their regenerative effects.^1^ Therefore, enhancing paracrine secretion capacity is a promising strategy to increase therapeutic benefits in MSC‐based therapies.

INPs could enable the transfection of therapeutic genes into MSCs, thus enhancing their paracrine secretion capacity. For example, peptide transfectant‐modified gold NPs successfully transfected MSCs with therapeutic genes and eventually enhanced brain‐derived neurotrophic factor (BDNF) expression in MSCs.^89^ In addition, iron oxide NPs such as ferrimagnetic iron oxide nanochains and cross‐linked iron oxide NPs could effectively transfect MSCs and significantly elevate the production of VEGF and BDNF (Figure 2C) from MSCs.^77^, ^90^ Moreover, treating with MSCs overexpressing BDNF significantly improved survival in mice with ischemic stroke injury, compared with naive MSCs (Figure 2D).^77^

The beneficial paracrine secretion of MSCs could also be enhanced without transfection, mainly through the biological effects of INPs themselves on MSCs.^91^ For example, the integration of iron‐containing NPs could make MSCs more potential restorative biological agents, since intracellular iron levels would affect cell function by regulating the expression of related genes.^113^ An iron‐releasing NP was designed to achieve endosome‐induced degradation and release of iron ions. Research confirmed that treatment of MSCs with appropriate concentrations of these NPs to induce mild ROS production can upregulate HIF‐1α expression, resulting in increased secretion of VEGF from MSCs. In the model of mouse skin wounds, transplantation of MSCs labeling the iron‐releasing NPs significantly enhanced angiogenesis and skin wound healing in comparison with naive MSCs.^75^ In addition, the property of iron oxide NPs to upregulate Cx43 expression can be utilized to promote the therapeutic benefits of MSCs. One study found that cardiac cells cocultured MSCs showed more active gap junction crosstalk between cells, and had greater potential in the treatment of myocardial infarction. Cx43 expression on MSCs and cardiomyocytes could be greatly upregulated by iron oxide NPs. The use of them would promote the gap junctional crosstalk between MSCs and cardiomyocytes and ultimately enhance the paracrine of MSCs for cardiac repair.^92^ These direct‐acting INPs have a relatively simpler structure than indirect‐acting gene carriers and can be more advantageous in terms of ease of preparation and quality control. However, their roles involve more links and more complex mechanisms. Further research is needed to obtain a unified paradigm for a wider range of applications.

### 2.4 INP integration enhances directed differentiation of MSCs

The directed differentiation of MSCs into injured tissue is another important mechanism of MSC‐based therapies for tissue repair.^114^ To improve the therapeutic benefits, transplanted MSCs need to differentiate into specific cell lines to adapt to different lesion sites.

INP integration can facilitate the directed differentiation of MSCs through providing mechanical stimulation. For example, the internalization of gold NPs could enhance the osteogenic differentiation of MSCs through the activation of some signaling pathways, such as p38 mitogen‐activated protein kinase (MAPK) pathway and the Wnt/β‐catenin signaling pathway.^93^, ^94^, ^95^ The intracellular mechanisms of gold NPs on MSC differentiation were further determined (Figures 2G–K). Gold NPs first bind with cytoplasmic proteins after endocytosis, causing mechanical stress. The subsequent activation of p38 MAPK pathway upregulates Runx2 and downregulates PPARγ. The osteogenic master transcription factor, Runx2, finally upregulates osteoblast markers, driving MSC differentiation to osteoblasts.^93^ In addition, the impact of gold NPs on MSC osteogenic differentiation has been demonstrated to be influenced by their size and shape.^115^ In comparison with rod‐shaped and star‐shaped, spherical gold NPs have shown greater potential in promoting osteogenesis. Gold nanospheres between 20 and 70 nm promote osteogenic differentiation of MSCs, of which 40 nm is a relatively optimal choice, while gold nanospheres below 10 nm inhibit osteogenesis and promote adipogenic differentiation.^116^ Internalizing iron oxide NPs could also induce similar mechanical stress, followed by the activation of the canonical MAPK signaling pathway, and eventually upregulate the expression of osteogenesis‐related genes of MSCs.^96^ In addition to the internalization process, MF could also provide mechanical stimulation to MSCs employing intracellular magnetic NPs.^106^, ^107^ For instance, MSCs integrated with magnetic NPs exhibited enhanced neuronal differentiation in the presence of an extremely low‐frequency electromagnetic field.^107^ Therefore, the introduction of MF would bring more possibilities for the future differentiation regulation of MSCs.^117^

Cargo‐carrying INPs can also lead the differentiation direction of MSCs through chemical stimulation. Silica NPs are the ideal candidate in this strategy, as they can enhance bone‐associated gene and protein expression through Si ion release even without carrying cargo.^97^, ^98^ Carrying differentiation‐inducing drugs can further enhance their role in the regulation of differentiation. For example, MSNs carrying bone morphogenetic protein‐2 (BMP‐2) peptide, dexamethasone, and some bioinorganic ions could effectively induce osteogenic differentiation of MSCs.^101^, ^102^ Iron oxide NPs bound with fibroblast growth factor 2 (FGF2) greatly enhanced the trilineage differentiation of MSCs.^109^

Direct introduction of differentiation‐related genes into MSCs via INP integration is another strategy to regulate the differentiation of MSCs. Research demonstrated that MSNs carrying the BMP‐2 gene^103^ or miR‐26a^104^ could encourage MSC osteogenic differentiation, and gold NPs carrying genes related to lipogenic differentiation can induce adipose regeneration.^108^

The target cell lines for directed differentiation of MSCs mainly focus on osteoblasts and also involve chondrocytes, adipocytes, and neuronal cells. Therefore, the range of target tissues that can be treated using the differentiation function of MSCs is limited. Moreover, studies on INPs stimulating the differentiation of MSCs have mostly remained at the stage of in vitro experiments. Their differentiation in vivo has not been fully investigated. In contrast, by promoting different paracrine factors secretion, MSCs can be applied to cure a broad range of illnesses, especially brain diseases. Therefore, focusing on the use of INPs to improve the paracrine function of MSCs may be a more promising strategy to improve efficacy.

## 3 INP‐INTEGRATED MSCS FOR ANTITUMOR THERAPY

MSCs are a “double‐edged sword” for tumor therapy.^118^ On the one hand, they may promote tumor growth by modulating immune surveillance, apoptosis, and angiogenesis.^119^ On the other hand, they may suppress tumor growth by blocking survival signals such as the Akt and Wnt pathways.^120^ However, it is not controversial that MSCs have a natural predisposition to the tumor microenvironment.^121^, ^122^ By taking advantage of this property and also equipping MSCs with tumor‐killing agents, MSCs can be used as a new tool for antitumor therapy. INP integration is the ideal approach to realize the above design. On the one hand, INPs could serve as carriers for various drugs or genes and increase the drug‐loading capability of MSCs. On the other hand, their self‐physical high thermal properties could exert a synergistic killing effect with the agent loaded. Therefore, INP‐integrated MSCs are potential tumor‐killing biological agents. The relevant contents are summarized in Table 2.

**TABLE 2:** Summary of the current role performed by the INPs in facilitating MSC‐based antitumor therapies.

Antitumor pattern	Nanoparticle	Form	Modification	Cargo/Effect	Integration approach	Extra feature	Subsidiary mean	Tumor type	Administration approach	References
Biochemical antitumor	MSN	Nanosphere	/	DOX	Endocytosis	/	/	NMU rat mammary cancer cells	In vitro coculture	^123^
	MSN	Nanosphere	Responsive copolymer p(MEO_2_MA‐co‐THPMA); PEI	DOX	Endocytosis	US responsiveness	US (1 MHz, 3 W/cm^2^, 5−10 min, continuous application)	NMU rat mammary cancer cells	in vitro coculture	^124^
	Silica nanorattle	Nanorattle	Mouse anti‐human CD90 or CD73 mAb	DOX	Anchor	/	/	U251 glioma tumor cells	Intratumoral injection	^58^
	Carbon nanotube	Nanotube	Anti‐CD90 Abs	DOX	Anchor	/	/	A549 lung tumor	*iv*	^59^
	Ferrimagnetic iron oxide nanochain	Nanochain	PEI	HSV‐tk/GCV suicide gene	Endocytosis	Upregulation of Cx43 expression	*ip* administration of GCV	C6 glioma cells	*iv*	^125^
	Magnetic core–shell NP composed of ZnFe_2_O_4_ core and mesoporous silica shell	Core–shell	PEI	Heat‐inducible TRAIL plasmid via HSP70B′ promoter	Endocytosis	Activation via mild magnetic hyperthermia (∼41°C); magnetically facilitated uptake	AMF (5 kA/m, 225 kHz)	A2780 human ovarian cancer cells	Intraperitoneal injection	^126^
	MnO_2_ NP	Nanosphere	/	Ce6 (Photodynamic effect)	Endocytosis	Efficient catalytic production of oxygen from H_2_O_2_ under acidic conditions	Laser (633 nm, 0.5 W/cm^2^, 3 min)	Lewis lung carcinoma cells	*iv*	^127^
Physical antitumor	PH‐sensitive gold NP	Nanosphere	Citraconic amide	Photothermal effect	Endocytosis	Aggregation in acidic endosome	Laser (660 nm cw, ca. 1 cm diameter, 0.5 W/cm^2^, 60 s)	HT‐1080 human fibrosarcoma cells	*iv*	^128^
	Gold NP/graphene oxide hybrid sheet	Nanosheet	α‐Synuclein protein (gold NP)	Photothermal effect	Anchor	Strong plasmon coupling between NPs via tight packing	Laser (808 nm cw, 1.5 W/cm^2^, 5 min)	HT‐1080 human fibrosarcoma cells	*iv*	^60^
Synergistic antitumor	Magnetic NP	Nanosphere	Glyceryl monooleate	PTX	Endocytosis	Magnetic hyperthermia	AMF (11 kA/m, 285 kHz, 15 min)	MAT‐LyLu prostate cancer	Intratumoral injection	^129^
	US‐responsive MSN	Nanosphere	PEI	CD:UPRT suicide fusion gene	Endocytosis	Potential to carry antitumor drug	/	NMU rat mammary cancer cells	in vitro coculture	^130^
	Gold nanorod‐embedded hollow periodic mesoporous organosilica nanosphere	Hollow nanosphere	/	PTX	Endocytosis	Photothermal effect	Laser (808 nm, 1.3 W/cm^2^, 5 min)	MCF‐7 breast cancer cells	Intratumoral injection	^131^
	Magnetic MSN with Fe_3_O_4_ core	Core–shell	PEI	Plasmid IL12 and photosensitizer MB	Endocytosis	Photodynamic effect and immune effect	Laser (660 nm, 0.5 W/cm^2^, 5 min)	EMT‐6 murine mammary cancer cells	*iv*	^61^
	SPION	Nanosphere	Carboxydextran	/	Endocytosis	Upregulation of EGFR expression to block EGF/EGFR signaling‐derived tumor growth	/	HT‐29 colon adenosarcoma cells	*iv*	^132^
	Ultrasmall gold nanocluster	Nanocluster	/	/	Endocytosis	Coradiotherapy	Lentiviral transfection of Egr1‐hNIS; ^131^I treatment	MDA‐MB‐231 triple‐negative breast cancer	*iv*	^133^

Abbreviations: Abs, antibodies; AMF, alternating magnetic field; CD:UPRT, cytosine deaminase and uracil phosphoribosyl transferase; Ce6, chlorin e6; DOX, doxorubicin; EGF, epidermal growth factor; EGFR, epidermal growth factor receptor; Egr1‐hNIS, early growth response protein1‐human sodium iodide symporter; GCV, ganciclovir; HSV‐tk/GCV, herpes simplex virus thymidine kinase/ganciclovir; *ip*, intraperitoneal injection; *iv*, intravenous injection; IL12, interleukin‐12; mAb, monoclonal antibody; MB, methylene blue; MSN, mesoporous silica nanoparticle; NP, nanoparticle; PEI, polyetherimide; PTX, paclitaxel; SPIO, super paramagnetic iron oxide; US, ultrasound.

### 3.1 INP‐integrated MSCs for biochemical antitumor

#### 3.1.1 INP integration mediates drug‐loading

Tumor‐killing agents can not only kill tumors but also diminish MSC functionality and decrease MSC vitality. Packaging antitumor drugs within NPs can avoid direct exposure thus reducing the potential adverse effects on cell vectors.^134^ MSNs are ideal candidates, as MSNs show strong drug‐loading capacity for different chemical molecules owing to their special porous structures and large surface areas.^135^ A previous study demonstrated that after integrated with doxorubicin (DOX)‐loaded NPs, MSCs begin able to induce the death of cocultured NMU cancer cells.^123^ And the study also confirmed that NP‐integrated MSCs retained their homing capacity toward the cancers in vitro and in vivo. However, DOX‐loaded NPs significantly reduced MSC viability 2 days after internalization. This demonstrates leakage of the drug loaded still happened in MSNs, due to their open pores. Therefore, MSNs need to be further modified. For example, MSNs could be covalently grafted with a US‐responsive copolymer to the surface to get US‐responsive NPs (UR‐NPs).^124^ The UR‐NPs were able to keep the cytotoxic agents inside, which ensured the survival of transporting MSCs and successfully released those cargoes when exposed to US. Therefore, MSCs could become more outstanding lethal biological agents that would minimize hurting innocents (including themselves) with the help of the modified MSNs.

In addition, INPs also could be anchored to MSC membranes utilizing specific antibody‐antigen recognition. A previous study conjugated silica‐nanorattles on the MSC membranes and successfully delivered the membrane‐conjugated nano drug in the skin‐xenograft mice model.^58^ These silica‐nanorattles were designed to be coupled with monoclonal CD73 or CD90 antibodies, allowing them to specifically bind to CD73 and CD90 antigens on the membranes of MSCs. Another research further extended the reach of MSC membrane‐conjugated therapies based on carbon nanotubes to deep lung tumor tissues.^59^ Besides, intracellularly loaded MSCs and membrane‐conjugated MSCs were compared in the research, which confirmed that membrane‐conjugated strategy had less impact on the innate functionality (e.g., homing ability) of MSCs and could bear more anticancer drugs. Despite the advantages of this strategy, it should be designed carefully since covering antigens on MSC membranes might alter the cellular function or fate of the MSCs.

Comparing the above two methods to prepare INP‐integrated MSCs, endocytosis is the more conventional and safer strategy. The cytotoxic agents loaded inside INPs are further encapsulated by cells. This could minimize exposure of cytotoxic drugs to the external environment during carrier transport. However, the endocytosis method may negatively affect vector function and have limited loading capacity. The anchoring approach could overcome these drawbacks but raise new problems. First, INPs attached are more susceptible to perturbations from external environments such as shear force, protein adsorption, and rapid clearance during transport, resulting in uncertain in vivo fate. Second, substantial masking of antigen‐binding sites on MSCs may cause potential harm to their function. Besides, further binding of INPs to antibodies increases the complexity of preparation. Therefore, extensive mechanistic studies are needed to optimize the anchoring strategy. And endocytosis is still the current predominant strategy to obtain INP‐integrated MSCs.

#### 3.1.2 INP integration mediates gene‐loading

Gene transfection is another way to prepare MSCs as lethal biological agents.^136^, ^137^, ^138^ MSNs with polyethyleneimine (PEI) coatings successfully transfected expression plasmids containing two suicide genes into MSCs. Therefore, the transfected MSCs were then able to induce the death of cocultured NMU tumor cells.^130^ In addition to cationic polymer modifications, polysaccharide modifications can also complement INPs for gene delivery, as these polysaccharides can effectively mediate receptor binding to facilitate stem cell uptake.^139^, ^140^

**FIGURE 3:**
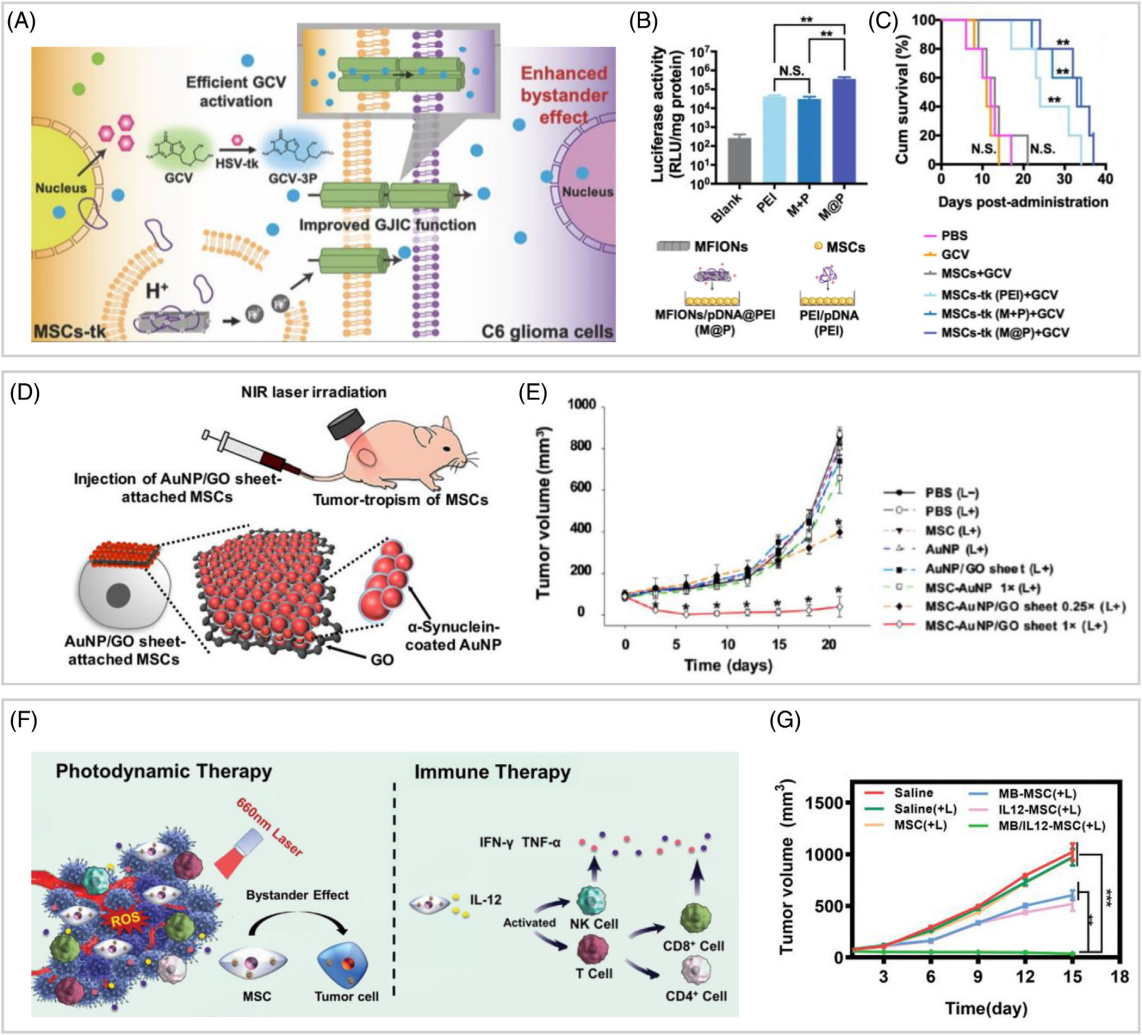
Representative strategies of INP‐integrated MSCs for antitumor therapy. (A) Schematic illustration of MFION‐integrated MSCs for cancer suicide gene therapy. (B) MFION‐integration had significantly higher transfection efficiency for MSCs. (C) MFION‐integrated MSCs significantly improved the median survival time of glioma rats. Reprinted with permission from Ref. 125. (D) Schematic illustration of an anchoring strategy to endow MSCs with gold NP aggregates for effective photothermal antitumor. (E) The gold NP aggregates attached MSCs significantly inhibited tumor growth during the 21‐day evaluation period. Reprinted with permission from Ref. 60, Copyright 2017 American Chemical Society. (F) Schematic illustration of the synergistic photodynamic and immunotherapeutic properties of MSCs integrated with MSN‐based nanocomplexes. (G) Treatment of MSCs with synergistic antitumor effects significantly inhibited tumor growth. Reprinted with permission from Ref. 61, Copyright 2022 Wiley‐VCH GmbH.

Apart from surface modification, inorganic nanocore is also critical. On the one hand, INPs themselves are a promising tool for gene delivery. For example, PEI‐modified ferrimagnetic nanochains achieved full expression of HSV‐tk in MSCs, showing significantly higher transfection efficiency than that of PEI‐based gene complexes (Figure 3B).^125^ High HSV‐tk expression in MSCs was able to convert nontoxic ganciclovir (GCV) into toxic metabolites (Figure 3A). Meanwhile, the nanochains composed of iron oxide triggered Cx43 overexpression in MSCs and facilitated intercellular communication between MSCs and tumor cells, thereby increasing the transfer of toxic metabolites to tumor cells and ultimately inducing cell death through enhanced bystander effects. As demonstrated in the rat model of glioma, ferrimagnetic nanochain‐transfected MSCs significantly ameliorated HSV‐tk/GCV suicide gene therapy with 40% more median survival time, compared with MSCs transfected with PEI (Figure 3C).

On the other hand, the properties of iron oxide NPs derive some advantages. First, their magnetic properties allow them to accelerate the deposition of their own and gene loaded on the cell membrane.^141^, ^142^ Second, enhanced intercellular communications mediated by iron oxide NPs might benefit the transportation efficiency of lethal gene products toward tumor cells.^125^ Third, integrated iron oxide NPs can achieve on‐demand responsive release, as they can generate heat when exposed to the alternating MF (AMF). For example, a magnetic core–shell NP achieved efficient delivery of heat‐inducible TRAIL plasmid to MSCs.^126^ Moreover, this specially designed NP was capable of mediating mild magnetic hyperthermia to initiate gene expression, and then effectively induced the death of ovarian cancer cells.

### 3.2 INP‐integrated MSCs for physical antitumor

Hyperthermia can induce cancer cell death at temperatures above 43°C.^143^ INPs such as gold NPs and magnetic NPs can serve as heat sources, so INP‐integrated MSCs are promising physical antitumor biologics. Published studies have confirmed that MSCs integrated with gold NPs could effectively treat cancer through photothermal therapy alone.^60^, ^128^ However, the effectivity of photothermal therapy depends heavily on the aggregation of gold NPs, as the aggregates have stronger resonance absorption. Endocytosis and anchoring are the two strategies to endow MSCs with gold NP aggregates. Critical to the endocytosis approach is to achieve effective enrichment of aggregates within MSCs. Kim and coworkers^128^ designed a pH‐responsive gold NP that could cluster together in acidic endosomes. Moreover, MSCs bearing the aggregates were shown to significantly slow the growth of tumor volume upon NIR laser irradiation on tumor‐bearing mice, compared with other irradiation groups including the naive MSC group.^128^ As for the anchoring approach, a gold NPs‐based hybrid sheet was developed by adsorbing and tightly arranging α‐synuclein protein‐coated gold NPs on both sides of a graphene oxide sheet (Figure 3D). This hybrid sheet could adsorb fibronectin from the surrounding medium and bind to integrin β1 on the MSC membrane, thus providing photothermal therapeutic capability to MSCs via membrane attachment. in vivo experiments showed that the sheet‐layer attached MSC group had a maximum temperature of 58.0°C after 5 min of NIR laser irradiation, compared with other laser irradiation groups including the conventionally internalized MSC group and the naive MSC group. Further experiments demonstrated that sheet‐layer attached MSCs significantly inhibited tumor growth during the 21‐day evaluation period while there was no significant difference in any of the other laser irradiation groups compared with the control group (Figure 3E).^60^ Yet at present, the successful application of magnetothermal monotherapy based on integrated MSCs for cancer treatment has not been reported. Effective antitumor treatment by physical hyperthermia alone is difficult unless it relies on a refined nanostructure design. Physical hyperthermia for adjuvant anticancer therapy is a more promising direction for future research.

### 3.3 INP‐integrated MSCs for synergistic antitumor

The diverse properties of INPs enable the integrated MSCs to exert multiple anticancer effects, thereby enhancing therapeutic efficacy.^144^

For INPs with multiple advantageous properties simultaneously, a single type of INP can confer the synergistic anticancer capacity to MSCs. For instance, magnetic iron oxide NPs have both self‐physical hyperthermia and drug‐carrying capacity, allowing for magnetothermal treatment in conjunction with chemotherapy. In an in vivo rat prostate tumor model, MSCs bearing magnetic NPs for simultaneous paclitaxel delivery and hyperthermia achieved better therapeutic outcomes with the greatest reduction in tumor volume and weight, compared with any monotherapy.^129^ Additionally, MSNs are the ideal candidate for codelivery of drugs and genes. Based on the previous study, Vallet‐Regí et al.^130^ further demonstrated that UR‐NPs could also be used as nonviral transfection agents for MSCs through PEI modification. The research suggested that UR‐NPs were a promising medium to prepare MSCs for synergistic antitumor. Moreover, a recent study successfully used MSN‐based nanocomplexes to achieve codelivery of the immune gene and photosensitizers to MSCs (Figure 3F). The integrated MSCs were able to continuously secrete interleukin‐12, resulting in a rapid increase of IFN‐γ and TNF‐α in the tumor microenvironment, leading to immune response activation. Furthermore, the inclusion of photosensitizers enabled the integrated MSCs to have synergistic photodynamic and immunotherapeutic properties, thereby exhibiting optimal antitumor activity in breast cancer models (Figure 3G).^61^

As for INPs with narrow advantageous properties, for example, gold NPs, the poor drug‐loading space limits the combination with chemotherapy. Therefore, it is necessary to use other materials to fill the gap. Lu and coworkers^131^ embedded gold nanorods in hollow silica shells to achieve synergistic chemo‐photothermal antitumor in MSC‐based therapies. Compared with pure photothermal therapy, the joint strategy significantly inhibited breast cancer tumor growth.^131^

In conclusion, INP‐integrated MSCs are expected to be developed as an excellent delivery platform for other anticancer therapies to exert more specific and effective antitumor effects.^61^ Chemotherapy, photothermal, photodynamic, and immunotherapy are widely used cancer treatments.^145^, ^146^, ^147^, ^148^, ^149^, ^150^ Despite the tremendous progress made, however, there are some limiting factors that hinder the widespread implementation of these therapies. For example, systemic toxicity and immune‐related adverse effects are among the major disadvantages of chemotherapy and immunotherapy,^151^ respectively. In the case of photothermal and photodynamic, monotherapy has limited efficacy, making complete eradication of solid tumors difficult and requiring a combination of other therapies.^152^ In addition, both therapies also have the potential to harm normal cells if the location and dose of the light source cannot be precisely controlled. Delivery platforms that can effectively deliver these therapies to the desired target cells can improve efficacy and reduce off‐target adverse effects.^153^, ^154^ MSCs have a natural predisposition to tumors,^121^, ^122^ and INPs are capable of loading mature antitumor agents into MSCs. Moreover, the ability of INPs to codeliver multiple drug types and their self‐physical high thermal properties can further enable combination therapies,^155^, ^156^ such as photothermal therapy with chemotherapy.^131^

## 4 INP‐INTEGRATED MSCS FOR SELF‐TRACKING

Stem cell therapy involves a wide range of mechanisms and its therapeutic principles are rather complex. At present, it is difficult to study the mechanism and most of the studies focus only on the efficacy level. Understanding the fate of transplanted MSCs in vivo is critical to the facilitation of mechanism research and further optimization of treatment strategies. Fluorescence imaging based on luciferase,^59^ fluorescent proteins,^125^ and fluorescently labeled antibodies^126^ are traditional cell‐tracking tools that are widely used to track the distribution of cells in vivo. Among them, in vivo fluorescence imaging enables real‐time tracking, but mostly provides a coarser image of organ‐level distribution. Tissue immunofluorescence staining and fluorescence confocal imaging provide distribution at the cellular level but only static information at a single time point. INP integration may enable real‐time tracking in stem cell therapy and provide relatively accurate information because it allows visualization of MSC groups in multiple noninvasive imaging modalities.^157^, ^158^, ^159^ The cell‐tracking applications of INPs in MSC‐based therapies are summarized in Table 3.

**TABLE 3:** Summary of the cell tracking application of INPs in MSC‐based therapies.

Purpose	Nanoparticle	Imaging mode	Animal model	References
Promotion of transplant accuracy	CoPP‐loaded MSN	PA	Nude mice percutaneous intramyocardial injection model	^86^
	Gold nanosphere	PA	Rodent spinal cord	^157^
	Wnt3a protein‐loaded and a cell‐penetrating peptide‐conjugated porous silicon NP	US	Healthy nude mice intramyocardial injection model	^87^
	Exosome‐like silica NP	US	Nude mice subcutaneous injection model	^160^
Long‐term longitudinal tracking	SPION	MRI (4 weeks)	Healthy mice intracerebral injection model	^158^
	Silica‐coated cobalt‐zinc‐iron NP	MRI (4 weeks)	Healthy rat intracerebral injection model	^161^
	Glucose‐MDDA‐coated gold NP	CT (1 month)	FSL depression rat model	^159^
	BSA blended and PLL layer modified gold NP	CT (23 d)	Pulmonary fibrosis injury mouse model induced by bleomycin	^162^
	PEG and glucose‐coated gold NP	CT (4 weeks)	Duchenne muscular dystrophy mouse model	^163^
Cell viability tracking	IR775c layered silica‐gold nanorod	PA	Normal mice gastrocnemius intramuscular injection model	^164^
Joint real‐time and long‐term tracking	Gd^3+^‐doped silica NP	US and MRI	Healthy nude mice intramyocardial injection model	^165^
	Silica‐iron oxide NP	US and MRI	Myocardial infarction murine model	^166^
Complementary anatomical and idiosyncratic information	Multi‐GNRs crystal‐seeded magnetic mesoporous silica nanobead	PA and MRI	Mice brain ischemia/reperfusion model	^167^
	^125^Iodine labeled silica coated‐SPIO NP	SPECT and MRI	Rat ischemic stroke model	^168^
	ICG‐loaded and PLL‐modified gold NP	NIRF and CT	Silica‐induced lung fibrosis mouse model	^169^

Abbreviations: BSA, bovine serum albumin solution; CoPP, cobalt protoporphyrin; CT, computed tomography; ICG, indocyanine green; MDDA, 12‐mercaptododecanoic acid; MRI, magnetic resonance imaging; MSN, mesoporous silica nanoparticle; Multi‐GNRs, multigold nanorods; NIRF, near‐infrared fluorescence; NP, nanoparticle; PA, photoacoustics; PEG, polyethylene glycol; PLL, poly‐l‐lysine; SPECT, single photon emission computed tomography; SPIO, super paramagnetic iron oxide; SPION, superparamagnetic iron oxide nanoparticles; US, ultrasound.

### 4.1 INP integration enables location tracking of MSCs

The rapid development of in vivo imaging modalities and nanotechnology has facilitated the emergence of diverse in vivo tracking techniques for MSCs. INP integration enables the visualization of MSCs in multiple imaging modalities, as INPs are themselves contrast agents for several imaging modalities. As for the other imaging modalities, they can also be used as carriers of corresponding tracers. In addition, MSNs could even widen the optical absorption of CoPP to the NIR region to fit PA imaging through intermolecular aggregation in mesopores.^86^ The characteristics of imaging modalities used in the self‐tracking of INP‐integrated MSCs are summarized in Table 4. Each of the modalities has its advantages and disadvantages. It is often necessary to select the appropriate imaging modality for effective tracking depending on the purpose of the study, and then design the matching NPs.

**TABLE 4:** Characteristics of imaging modalities used in the self‐tracking of INP‐integrated MSCs.

Imaging features	Imaging modality	Input signal	Tracer	Spatial resolution	Temporal resolution	Penetration depth	References
High specificity	Photoacoustics	Light	Gold NPs	5 μm–1 mm (depth dependent)	s–min	<6 cm	^157^
	Single photon emission computed tomography	Radionuclide (γ‐rays detected)	Radioisotopes (INPs as carriers)	0.5–10 mm	min	Unlimited	^168^
	Near‐infrared fluorescence	NIR light	NIR light dyes (INPs as carriers)	2–3 mm	s–min	<2 cm	^169^
Offer anatomical depiction	Ultrasound	Sound waves	Silica NPs	10 μm–2 cm (depth dependent)	s–min	10 ms	^160^
	Magnetic resonance imaging	Radiofrequency	Iron oxide NPs	25 μm–1 mm	min–h	Unlimited	^158^
	Computed tomography	X‐rays	Gold NPs	25 μm–1 mm	s–min	Unlimited	^159^, ^162^, ^163^

Abbreviations: CT, computed tomography; INP, inorganic nanoparticle; MRI, magnetic resonance imaging; NIR, near‐infrared; NIRF, near‐infrared fluorescence; PA, photoacoustics; SPECT, single photon emission computed tomography; US, ultrasound.

**FIGURE 4:**
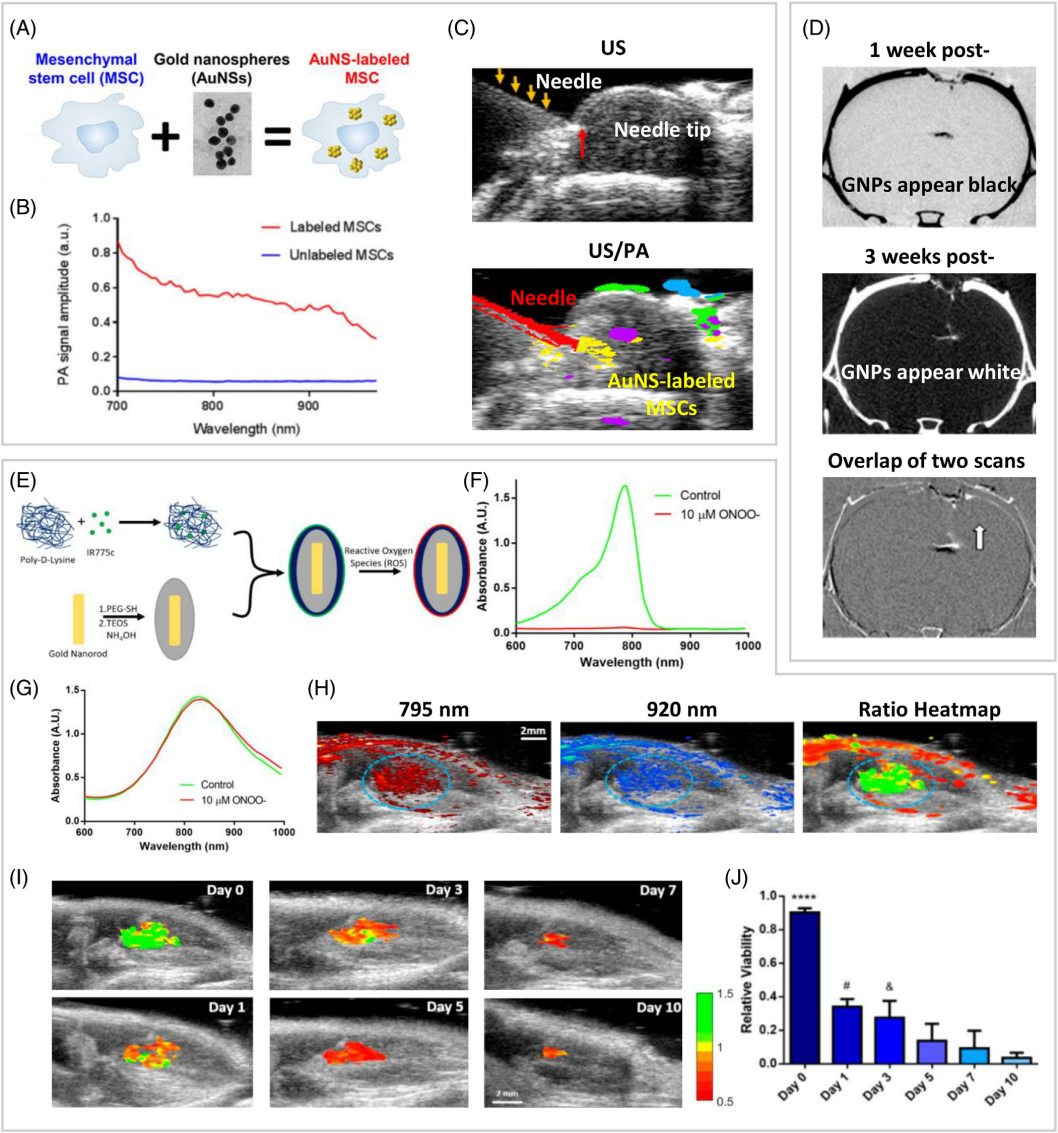
Representative examples of INP‐integrated MSCs for in vivo self‐tracking. (A) Schematic illustration of the preparation of gold nanosphere‐labeled MSCs. (B) The PA spectrum analysis of gold nanosphere‐labeled MSCs and unlabeled MSCs. (C) Visualization of needle placement via US imaging and distribution of gold nanosphere‐labeled MSCs via US/PA imaging in rodent spinal cord. Reprinted with permission from Ref. 157, Copyright 2018 American Chemical Society. (D) Long‐term tracking indicated position changes of gold NP‐integrated MSCs. Reprinted with permission from Ref. 159, Copyright 2014 American Chemical Society. (E) Schematic illustration of the synthesis process of IR775c layered silica‐gold nanorods. (F) The PA signal of IR775c decreased significantly by peroxynitrite. (G) The PA signal of gold nanorods was generally unaffected by peroxynitrite. (H) The availability of a heatmap of 795 nm/920 nm based on PA images of transplanted MSCs (marked by blue circle) at 795 and 920 nm on day 0. (I) Heatmaps for each time point. (J) The quantified relative viability of MSCs at each time point. ****p < 0.0001 compared with all other days. Reprinted with permission from Ref. 164, Copyright 2019 American Chemical Society.

Topical administration is also used for stem cell delivery in treatment. Greater precision is required in the case of topical delivery to critical organs such as the heart. PA and US are ideal imaging modalities to provide accurate real‐time guidance and help confirm the success of implantation to ensure adequate cell delivery, as they have high temporal resolution.^170^, ^171^ Among them, PA is usually used to determine the location of the implanted cells, while US is more often used to provide an anatomical description.^86^, ^159^ For example, gold nanospheres coupled with PA could timely feedback on the location of labeled MSCs. When combined with the satisfactory anatomical description provided by US, this method guided the precise delivery of MSCs to the target location in the spinal cord and visualized the entire infusion process (Figures 4A–C).^157^ Current studies rarely use US to track the location of INP‐integrated MSCs, as US is susceptible to interference from the surrounding environment. Reforming the structure of tracers to enhance acoustic imaging capabilities is a potential approach to increase the recognition of US imaging.^87^, ^172^ For instance, an exosome‐like silica NP (ELSN) was confirmed to have stronger echogenicity. Further in vivo experiments showed that ELSN‐integration enhanced in vivo echogenicity of MSCs by 3.3 times and increased the sensitivity of MSCs toward US imaging with a detection limit of 475 cells.^160^

To promote the research of MSC therapeutic mechanisms, long‐term longitudinal tracking of implanted MSCs is necessary. MRI and CT are widely used for long‐term tracking, as their penetration depth is unlimited.^173^, ^174^ For example, long‐term tracking of MSCs integrated by iron oxide NPs transplanted in the mouse brain using MRI showed that a small number of MSCs could still be tracked at the fourth week after implantation.^158^ Similarly, gold NP integration coupled with CT imaging could enable the in vivo tracking of integrated MSCs for more than 20 days.^159^, ^162^ Using these techniques to track MSCs longitudinally has the potential to help clarify therapeutic mechanisms. Several studies have conducted preliminary explorations. For example, long‐term CT imaging of gold NP‐integrated MSCs revealed their specific migration to depression‐associated brain regions in model rats (Figure 4D). The researcher further hypothesized that MSCs have a key role in improving depressive symptoms.^159^ In addition, after topical administration in a Duchenne muscular dystrophy mouse model, CT imaging results showed long‐term residence of MSCs in the muscle and local recovery of muscle calcification. The phenomenon demonstrated the benefit of MSCs to treat this type of muscle disease.^163^

Furthermore, multimodal imaging can combine the advantages of different imaging modalities and provide more reliable tracking information on MSCs. INP integration can realize multimodal imaging with relative ease, due to their inherent imaging properties and great cargo‐carrying capacity. Meanwhile, INP integration also avoids some of the drawbacks associated with multistep tagging strategies, such as time consumption and difficulty in quantifying tagging efficiency.^175^ Depending on the requirements of research, the strategies for multimodal imaging can be broadly classified into two types. One is to combine an imaging modality for real‐time tracking with one for long‐term longitudinal tracking.^165^, ^166^ The other is to pair an imaging modality of high specificity with one offering anatomical depiction.^167^, ^168^, ^169^

### 4.2 INP integration enables viability tracking of MSCs

Determination of the transplanted MSC viability is also important for therapeutic applications. INP integration is a potential tool to visualize the in vivo viability of MSCs. Considering the negative correlation between intracellular ROS and cell viability, Suggs and coworkers^164^ invented a ROS‐sensitive photoacoustic dye IR775c layered silica‐gold nanorod (Figure 4E). In this system,^164^ the PA signal of IR775c at 790 nm decreases with increasing ROS concentration, while the PA signal generated by gold nanorods at 920 nm is unaffected by the ROS concentration in the surrounding medium (Figures 4F and G). Thus, the ratio of PA signals at 795 and 920 nm in each pixel can reflect the state of MSCs there (Figure 4H). Moreover, the relative viability of transplanted MSCs can be visualized by developing a ratio heatmap of PA signals (Figure 4I). Longitudinal viability tracking of muscle transplant MSCs in normal mice models via this system showed notable cell death within 24 h and an estimated 5% viability after 10 days (Figure 4J). Although the feasibility of the idea has been initially validated, it is still in the proof‐of‐concept stage. The dream of monitoring the survival of transplanted MSCs in clinical practice is still far from being realized.

## 5 INP‐INTEGRATED MSCS FOR SIMULTANEOUS TREATMENT AND TRACKING

Single inorganic nanomaterials with multiple functionalities, allowing for the creation of “all in one” tagged MSCs agents. They achieve the improvement of stem cell therapy effectiveness and precise tracking without increasing the complexity of modification.

First, INP‐integrated MSCs can achieve both regenerative and imaging functions. For instance, the tri‐functional INP was created via incorporating insulin‐like growth factors (IGFs) and SPIONs into large pores of mesoporous foam silica NPs.^166^ Among them, SPIONs enhanced the MRI contrast of cells and allowed magnetic manipulation, while silicon‐based carriers increased cellular US contrast and allowed sustained release of the prosurvival agent IGFs. in vivo and in vitro experiments demonstrated that MSCs integrated with the tri‐functional INPs possessed dual‐modal US/MRI tracking capabilities, enabling real‐time guided injection and long‐term in vivo follow‐up. Meanwhile, the accessibility of prosurvival agent release and magnetic manipulation promoted MSCs survival and retention, respectively. Therefore, the integrated MSCs have the potential to address the challenges of injection errors, low survival rates, and low retention rates encountered in cell transplantation. The integrated MSCs exhibited superior therapeutic efficacy in comparison with the nonintegrated MSCs on a mouse model of ligation/reperfusion injury. In addition, there are numerous comparable “all in one” designs available to facilitate accurate transplantation of stem cells and to promote their survival in vivo. The utilization of a Wnt3a protein‐loaded porous silicon NP that possesses both US imaging and antioxidant protection for integration increased the precision of stem cell transplantation and improve cell survival rates.^87^ And integrating MSNs loaded with both CoPP and ^125^I into MSCs enabled bimodal PA/SPECT imaging, while simultaneously protecting the MSCs from oxidative stress via CoPP release.^176^ As for systemic administration, adequate delivery to the lesion site and long‐term follow‐up are necessary. A multigold nanorods crystal‐seeded magnetic mesoporous silica nanobead was developed to label MSCs for enhancing homing via magnetic guidance and offering dual‐modality PA/MRI imaging.^167^

Second, INP‐integrated MSCs can also be designed for simultaneous antitumor therapy and tracking. An example study utilized iron oxide nanoclusters as a framework and assembled gold nanorods on its surface to form plasmonic‐magnetic nanostructures loaded with DOX.^177^ The integration of hybrid NPs into MSCs promoted their tropic migration to tumor cells by upregulating CXCR4 on the cell surface. Additionally, DOX and gold nanorods allowed for chemo‐photothermal treatment, while gold nanorods also enabled photoacoustic imaging in these integrated MSCs. And the integrated MSCs showed superior antitumor efficacy via chemoradiotherapy in comparison with other treatment groups in a nude mouse model of triple negative breast cancer. Similarly, a PTX‐loaded and gold nanorod‐embedded hollow silica‐based nanosphere was developed to endow MSCs with synergistic chemotherapeutic‐photothermal killing efficacy, as well as PA imaging capabilities.^131^

Imaging‐guided on‐demand drug release could dramatically improve the efficiency of drug delivery by MSCs. Previous studies have explored the use of US and magnetothermal techniques to control drug release or activate therapeutic gene expression.^124^, ^126^ Also, a proof‐of‐concept study has already been conducted to demonstrate the external control of therapeutic agent release from stem cells using light‐based methods.^177^ Plasmonic‐magnetic NPs could undergo photo‐controlled disintegration and control the drug release. Building on this knowledge, there is still potential for improving the current multifunctional nanoplatform for MSCs by incorporating an on‐demand drug release function.

However, the single‐component INP structure may not fully meet the diverse clinical needs. Most of the currently developed multifunctional NPs contain two or more inorganic nanomaterials, or carry multiple cargoes.^178^, ^179^ The clinical translation of multifunctional NPs is limited due to the complexity of their preparation process or unsatisfactory performance in a single function.^180^ A recent study successfully utilized a straightforward solvent evaporation‐driven technique to achieve the self‐assembly formation of a multifunctional nano‐agent with multiple components.^181^ And the study demonstrated that the resulting NPs effectively integrated CT imaging, magnetic manipulation, and long‐term antioxidant stress functions into the labeled MSCs.

In conclusion, the field multifunctional tagged agents for optimizing MSC function rapidly advanced due to the increasing demand for high‐quality stem cell therapy. A promising future approach is the development of multifunctional nano‐agents with simpler components or simpler synthesis methods,^182^ which is benefit for clinical translation of tagged MSCs.

## 6 FUTURE PERSPECTIVE

INP‐integrated MSCs would be a potential biological agent for multifaceted applications. On the one hand, they show great potential in regenerative therapies to treat a wide range of diseases and be traceable in multiple imaging modalities. Compared with other regenerative therapies such as growth factors, biomaterials, and gene therapy, INP‐integrated MSCs can not only serve as an excellent delivery platform for drugs, but also have ability to replace damaged or dysfunctional cells, and produce long‐term effects. On the other hand, the natural targeting capacity and enhanced drug‐loading efficiency of INP‐integrated MSCs can easily enable combination therapy for cancer. Several studies have confirmed the advantages of MSC‐based targeting over enhanced permeability and retention‐based NP targeting in terms of tumor targeting and penetration. Until now, it is seldom reported the comparison between actively targeted NPs and MSC‐based targeting platforms. In addition, INP‐integrated MSCs have higher biocompatibility than synthetic nano's platforms, and achieve unique anticancer therapies that rely on the function of living cells, such as gene therapy. As a result, therapies based on INP‐integrated MSCs can be developed into a large therapeutic platform for a multitude of refractory diseases.

Despite the outstanding progress of INP‐integrated MSCs in a variety of therapeutic applications, a number of important challenges remain to be addressed before they can be further translated into clinical applications. On the one hand, INP‐integrated MSCs necessarily inherit part of the challenges faced by INPs or MSCs. First, the biodegradation of INP systems is one of the key issues that hinder their wide use.^183^ Developing biodegradable INP systems with desirable physicochemical properties is the solution. Recently, 2D silicon/silicon nanosheets have gained attraction due to their high biodegradability, unique nanostructure, physicochemical properties, and biological effects, making them ideal for safe use in vivo and multifunctional biomedical applications.^184^ Understanding the degradation behavior of MSNs, particularly the relationship between their structure and dissolution, can facilitate adjusting nanostructure design to better achieve therapeutic goals.^185^ In regards to MSCs, a major challenge is their potential to have protumor effects, which raises concerns regarding their use in cancer therapy.^186^ Future strategies for the controlled elimination of transplanted cells would be helpful. Another challenge is the limited comprehension of the interaction between stem cells and the immune system, which may result in the rejection of transplanted cells.^73^ The presence of intracellular glycyrrhizin (GL) has been shown to inhibit the release of damage‐associated molecular pattern (DAMP) protein after cell injury, thereby reducing the inflammatory response of transplanted cells. Therefore, delivery of GL into MSCs using INPs is a promising strategy to mitigate immune rejection.^187^ On the other hand, conventional endocytosis tools have limited labeling efficiency and a short labeling lifetime, which may hinder the potential benefits of INP‐integrated MSCs.^188^, ^189^ Although, cell‐penetrating peptides^190^ and emerging bio‐orthogonal labeling means^191^, ^192^ have been utilized for INP surface modification to achieve higher integration efficiency. However, the potential cytotoxicity and complex chemical processes limit its clinical application.^193^ The development of effective clinical stem cell therapies requires an efficient and safe labeling strategy. The ferrimagnetic vortex‐domain iron oxide nanoring (FVIO) is a novel nanomaterial with exceptional magnetic properties. Recently, researchers have developed an advanced magnetic thermal labeling method based on FVIOs and achieved safe and effective labeling of MSCs, as they can locally induce heat to enhance membrane permeability.^63^ Furthermore, there has been a report on a microfluidic‐based technique for fast and effective labeling of stem cells.^194^ These methods show great potential for future clinical use.

Although as a novel biological agent, INP‐integrated MSCs are not yet approved for clinical trials, the MSCs and INPs that make up the system are being studied extensively in clinical trials, respectively.^195^, ^196^, ^197^, ^198^ There are currently over 1,500 clinical trials involving MSCs on ClinicalTrials.gov. MSCs have been shown to be effective in treating a variety of diseases, including lung and cardiovascular diseases.^199^, ^200^ In addition, large number of preclinical studies and clinical trials have demonstrated that MSC‐based therapies are safe.^23^, ^201^, ^202^ Furthermore, a few MSC‐based therapies have received regulatory approval in Canada and Europe.^186^ As for the most commonly utilized INPs in this review, although some of their applications not yet involved in drug delivery, have undergone substantial clinical research.^203^ For instance, total thermal ablation of prostate tumors has been demonstrated to be possible using nanoshells made of silica and gold and covered with (poly)ethylene glycol.^204^ Additionally, a hybrid silica NP for tumor imaging in melanoma patients has been cleared for clinical studies.^205^ More encouragingly, the United States Food and Drug Administration has approved several iron oxide NPs, including Feraheme and Injectafer, for anemia treatment due to a lack of iron.^206^ Among them, ferumoxytol (trade name Feraheme) has also been approved for clinical trials as an MRI contrast agent. Therefore, INP‐integrated MSCs have a great chance of entering clinical practice.

Despite these challenges, with continued advances in nanotechnology, imaging techniques, materials science, and cell labeling technology, we believe that INP‐integrated MSCs are a very promising biological agent for clinical applications.

## AUTHOR CONTRIBUTIONS

*Writing—original draft preparation*: Juan‐Juan Zheng. *Writing—reviewing and Editing, conceptualization and supervision*: Xin‐Chi Jiang. *Writing—reviewing and editing*: Yao‐Sheng Li. *Conceptualization and supervision*: Jian‐Qing Gao. All of the authors have read and approved the final manuscript.

## CONFLICT OF INTEREST STATEMENT

The authors declare no competing interests.

## ETHICS STATEMENT

Not applicable.

## Data Availability

Not appliable.
